# Oak Root Response to Ectomycorrhizal Symbiosis Establishment: RNA-Seq Derived Transcript Identification and Expression Profiling

**DOI:** 10.1371/journal.pone.0098376

**Published:** 2014-05-23

**Authors:** Mónica Sebastiana, Bruno Vieira, Teresa Lino-Neto, Filipa Monteiro, Andreia Figueiredo, Lisete Sousa, Maria Salomé Pais, Rui Tavares, Octávio S. Paulo

**Affiliations:** 1 Plant Systems Biology Lab, Center for Biodiversity, Functional and Integrative Genomics, Faculty of Sciences, University of Lisbon, Lisbon, Portugal; 2 Center for Environmental Biology, Faculty of Sciences, University of Lisbon, Lisbon, Portugal; 3 Plant Functional Biology Centre, Center for Biodiversity, Functional and Integrative Genomics, University of Minho, Braga, Portugal; 4 Department of Statistics and Operational Research, Center of Statistics and Applications from Lisbon University, Faculty of Sciences, University of Lisbon, Lisbon, Portugal; University of Nebraska-Lincoln, United States of America

## Abstract

Ectomycorrhizal symbiosis is essential for the life and health of trees in temperate and boreal forests where it plays a major role in nutrient cycling and in functioning of the forest ecosystem. Trees with ectomycorrhizal root tips are more tolerant to environmental stresses, such as drought, and biotic stresses such as root pathogens. Detailed information on these molecular processes is essential for the understanding of symbiotic tissue development in order to optimize the benefits of this natural phenomenon. Next generation sequencing tools allow the analysis of non model ectomycorrhizal plant-fungal interactions that can contribute to find the “symbiosis toolkits” and better define the role of each partner in the mutualistic interaction. By using 454 pyrosequencing we compared ectomycorrhizal cork oak roots with non-symbiotic roots. From the two cDNA libraries sequenced, over 2 million reads were obtained that generated 19552 cork oak root unique transcripts. A total of 2238 transcripts were found to be differentially expressed when ECM roots were compared with non-symbiotic roots. Identification of up- and down-regulated gens in ectomycorrhizal roots lead to a number of insights into the molecular mechanisms governing this important symbiosis. In cork oak roots, ectomycorrhizal colonization resulted in extensive cell wall remodelling, activation of the secretory pathway, alterations in flavonoid biosynthesis, and expression of genes involved in the recognition of fungal effectors. In addition, we identified genes with putative roles in symbiotic processes such as nutrient exchange with the fungal partner, lateral root formation or root hair decay. These findings provide a global overview of the transcriptome of an ectomycorrhizal host root, and constitute a foundation for future studies on the molecular events controlling this important symbiosis.

## Introduction

In nature, the roots of trees are engaged in a mutualistic association with soil fungi, called ectomycorrhizas (ECM). ECMs are abundant on temperate and boreal forests, where soil-borne fungi like truffles, boletes, amanitas and chanterelles colonize the roots of dominant tree species such as oak, pine, poplar, birch, eucalypt or aspen [1]. In this association, which dates back to 120 million years [2], the ECM fungus actively transfers nutrients and water to the host plant. In return, the plant can transfer up to 1/3 of the photosynthetically derived sugars to the fungus [3]. This exchange of metabolites is essential for the persistence of both tree and fungal mycelium, mainly in nutrient-poor soils, ECMs being one way to overcome nutrient and carbohydrate limitations faced by trees and fungi in forest ecosystems [3]. ECMs also play an essential role in the protection of trees from pathogens and from adverse abiotic conditions, like water stress or soil pollution [1]. In ECMs, the fungal mycelium forms a sheath around the short roots, called the mantle, isolating them from the surrounding soil. From the mantle, hyphae penetrate the root apoplast forming a net (the Hartig net) around the epidermal cells and sometimes the cortex cells, where nutrients are exchanged between partners. The fungal mycelium also extends into the soil forming a highly ramified network that contributes to increase the absorbing surface area of the root, since the fungus is able to explore and absorb nutrients from a greater volume of soil than could be exploited by the root alone. The development of ECM symbiosis is a highly regulated process involving morphological and physiological changes, including stimulation of lateral root development [4], increased root cell volumes [5], suppression of root hair formation [6] or enhanced photosynthetic efficiency [3]. Technological developments in plant genomics, particularly microarrays and EST sequencing, led to the identification of plant and fungal genes that are activated or repressed during ECM symbiosis in several host-fungal combinations [5], [7]–[10]. Similar expression profiles for several genes in different ECM plant-fungal combinations have highlighted many cellular functions that are regulated upon ECM development. In the plant partner, transcriptome analysis has revealed increased plant cell metabolism, activation of biotic and abiotic stress response, increased cell wall loosening, increased nitrogen transport and long-term down regulation of phosphorus sensing pathways and phosphorus uptake in roots [5]. Detailed information on the molecular processes operating in ECM host trees is relevant owing to their ecological significance, the economic importance of the species involved and the interest in exploiting this symbiosis to maximize tree productivity and sustainability. The generalization of genomic tools like next-generation DNA sequencing is increasing the number of organisms with available sequenced genomes, including several ECM fungal species, such as L*accaria bicolor*, *Tuber melanosporum*, as well as host trees like *Populus trichocarpa*. This sequencing data constitutes a fundamental resource for comparative genomic analysis and for discovery of genes of ecological interest. The recent release of the genomes of the ECM fungi *L. bicolor* and *T. melanosporum* is providing highly valuable information about this symbiotic lifestyle [11], [12]. However, plant transcriptomic response studies are still limited to species with fully-sequenced genomes, for which whole-genome arrays are available, such as *P. trichocarpa* [4], [5]. The rapid development of next-generation sequencing technologies can overcome this limitation, offering a unique opportunity for genomics and functional genomics studies in non-model organisms. Wide transcriptome analysis of additional ECM fungi and their host plants can help us to further characterize the molecular events occurring in symbiotic tissues by finding common gene networks that can be used to predict putative plant and fungal “symbiosis toolkits” and better define the role of each partner in the mutualistic interaction. The cork oak ESTs consortium (www.corkoakdb.org) was organized to develop genomic resources for cork oak (*Quercus suber*) to help address major items related to the understanding of the plant's adaptation processes to both biotic and abiotic factors, cork differentiation, ecophysiological interactions and interspecific hybridization and gene flow (Pereira-Leal et al. in press). In this context, we present here the results derived from a transcriptomic analysis of cork oak ECM root tissue performed by 454 pyrosequencing technology. Our goal was to elucidate the response of the host plant to ECM colonization by comparing symbiotic and non-inoculated roots. The experiment has yielded information regarding the identity of 2238 genes which are either up- or down-regulated as part of the response to ECM establishment. The genetic resources presented here constitute a major advance for studies on ECM symbiosis and will promote comparative genomics studies among ECM host plants.

## Results and Discussion

A *Pisolithus tinctorius* fungal innoculum was used to establish ECMs in cork oak roots in the greenhouse. The presence of distinct mycorrhizal roots was observed 3 weeks after inoculation; upon this time-point inoculated roots presented the typical morphotype of *P. tinctorius* mycorrhizae [13] (Fig. S1). Previous studies have shown that inoculation of cork oak with *P. tinctorius* increases plant performance, ECM plants having increased leaf area, increased nitrogen content, higher photosynthetic capacity and water use efficiency, when compared to non-inoculated plants [14]. For covering early and mature stages of interaction, RNA was extracted from inoculated plants at 1, 3, 8 and 16 weeks after inoculation. Two cDNA libraries were prepared for sequencing: an ECM root library prepared with RNA from mycorrhizal roots and composed of a mixture of transcripts from cork oak and *P. tinctorius*, and a control (non-symbiotic) library prepared from non-inoculated plants.

### Sequencing and assembly

The cDNA from both libraries was sequenced using 454 GS-FLX resulting in 2,292,583 raw reads. A status summary of the generated sequencing data is presented in Table 1. Before assemblage, the reads were passed through several quality control filters including the removal of low quality reads, trimming of adaptor/primer sequences, removing reads corresponding to chloroplast and mitochondrial genes and also reads from organisms predicted to be present as “contaminants”, such as the ECM fungus *P. tinctorius* used for establishing ECM roots, and a variety of soil organisms, like fungi, bacteria and virus. As expected the number of “contaminating” reads was higher in the ECM root library, with *P. tinctorius* reads contributing to the majority of these reads. This percentage of “contaminating” reads likely reflects the soil biodiversity of our conditions since cork oak plants were grown in an environment close to natural conditions, in non-sterilized soil in a greenhouse. After filtering, a total of 2,100,288 processed reads (92% of total raw reads) with an average length of 500 bp were obtained. The length distribution of these reads showed that most of them (81%) were more than 300 bp in length (Fig. S2).

**Table 1:** Summary of 454 data generated for cork oak root transcriptome and quality filtering.

Library	Total bp sequenced^a^	Total reads^b^	Low quality reads^c^	Primer/adaptor reads^d^	Short reads^e^	Chl. reads^f^	Mit. reads^g^	*P. tinctorius* reads^h^	Fungi, bacteria, virus reads^i^	High quality reads^j^	Average length reads^k^
ECM root	402663515	1044191	8	1277	74799	478	346	20614	1383	945286	500
non-symbiotic root	472006235	1248392	7	1328	88740	519	356	51	2383	1155002	512

a Total number of base pairs sequenced for each tissue sample. ^b^Total number of reads for each tissue sample. ^c^Number of low-quality reads removed. ^d^Number of trimmed reads containing primer/adaptor sequence. ^e^Number of short reads (less than 100 bp) removed. ^f^Number of reads identified as chloroplast reads. ^g^Number of reads identified as mitochondrial reads. ^h^Number of reads identified as *P. tinctorius* reads. ^i^Number of reads identified as fungi, bacteria and virus reads. ^j^Number of high-quality reads used for further analysis. ^k^Average length of high quality reads in bp.

The processed reads from both cDNA libraries were combined and assembled together using MIRA assembler. A status summary of the 454 sequencing data assembly is presented in Table 2. MIRA assembled a total of 1,931,868 reads (92% of the total reads after filtering), yielding 127,489 assembled sequences (contigs). The contigs obtained had an average length of 689 bp and 15% of them were more them 1000 bp in length. The length distribution of the contigs showed that most of them (85%) were more than 400 bp in length (Fig. S3).

**Table 2:** Summary of the 454 sequencing data assembly.

Reads after filtering^a^	Assembled reads^b^	Contigs	Average length of contigs (bp)	Large contigs^c^ (>1000 bp)	Max. length contig^d^ (bp)
2100288	1931868	127489	689	19195	7958

a Number of reads used for assemblage. ^b^Number of reads assembled into contigs. ^c^Number of contigs longer than 1000 bp. ^d^Length of the longest contig.

### Functional Annotation

As cork oak plants used for RNA extraction were mycorrhized with *P. tinctorius* and were grown in non-sterile soil under greenhouse conditions we expected a high degree of “contamination” with sequences from *P. tinctorius* and several other soil organisms. To further identify those “contaminating” sequences we performed a taxonomical assignment for the 127489 assembled contigs using two approaches: (1) the MG-RAST server and (2) a BLASTX search against Streptophyta (green plants) sequences deposited in the NCBI database. MG-RAST identified 55095 non redundant annotated protein features, with 34212 contigs assigned to the Streptophyta phylum. A taxonomic distribution of the assembled 454 data at the phylum level is shown in Figure S4. The most abundant taxonomic group was the Streptophyta phylum followed by the fungal groups, Basidiomycota (*P*. *tinctorius* belongs to the Basidiomycota) and Ascomycota, as expected. Other identified taxonomic groups include Chordata, Proteobacteria, Arthropoda and Nematoda, among others. BLASTX search against NCBI nr protein database retrieved 116133 hits that corresponded to 77375 unique accessions. From these, 39335 accessions had a Streptophyta annotation. Only the sequences classified as Streptophyta by the two approaches were considered for further analysis. These comprised a set of 19552 unique cork oak root putative sequences (Table S1). Using BLAST2Go we were able to annotate a total of 18802 sequences. All sequences matched Streptophyta proteins confirming that our analysis pipeline was efficient in removing “contaminating” organisms from the cork oak roots transcript set. The species that provided most of the top hits in BLAST nr searches was *Punus persica* followed by *Vitis vinifera* and *Populus trichocarpa*; *Arabidopsis* was found in the 12^th^ position (Fig. S5). Comparison of our cork oak root unigene set with EST collections from other oak species showed that a high percentage of our sequences had homologues on those databases, with 12715 unigenes (65%) mapping on the TIGR oak gene index (*Q. petraea* and *Q. rubor*) and 14214 unigenes (73%) on the 454 oak (*Q. alba* and *Q. rubra*) transcript sequences from the Fagaceae genomics web. Unmapped sequences could represent transcripts that are mainly expressed upon interaction with microorganisms since these databases are derived from unchallenged plant tissues.

### Comparison of transcriptome between ECM and non-symbiotic roots

**Figure 1:**
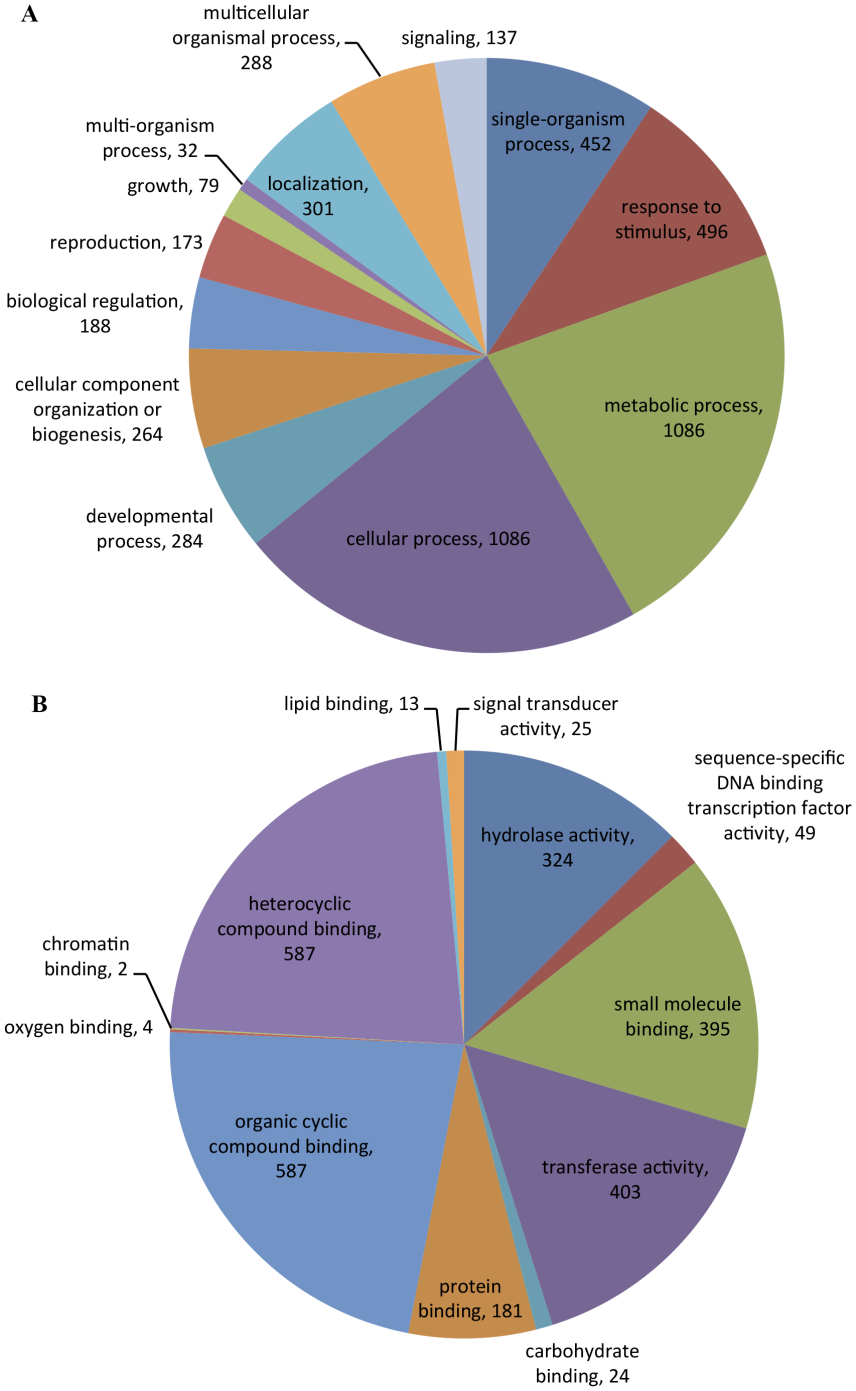
GO terms of differentially expressed genes in ECM roots versus non-symbiotic roots within the category of biological process (A) and molecular function (B).

To identify the molecular processes that are activated or repressed in roots by signals released by the ECM symbiotic fungus, the cork oak root unigene sequences were used for analysis of transcript abundance in ECM versus non-symbiotic roots. Using the edgeR approach we identified 2238 sequences with differential transcript abundance in ECM roots when compared to non-symbiotic roots (adjusted p-value ≤0.001 and absolute fold change ≥2) (Table S2). These sequences were classified into different functional categories using the plant-specific GO slims, which are a list of high-level GO terms providing a broad overview of the ontology content (Fig. 1). Within the biological process category, metabolic process, cellular process and response to stimulus were among the most highly represented groups. Organic cyclic compound binding and heterocyclic compound binding were the top groups in the GO category molecular function. From these differentially expressed genes (DEGs), 1198 showed higher expression in ECM roots and 1040 showed lower expression.

**Figure 2:**
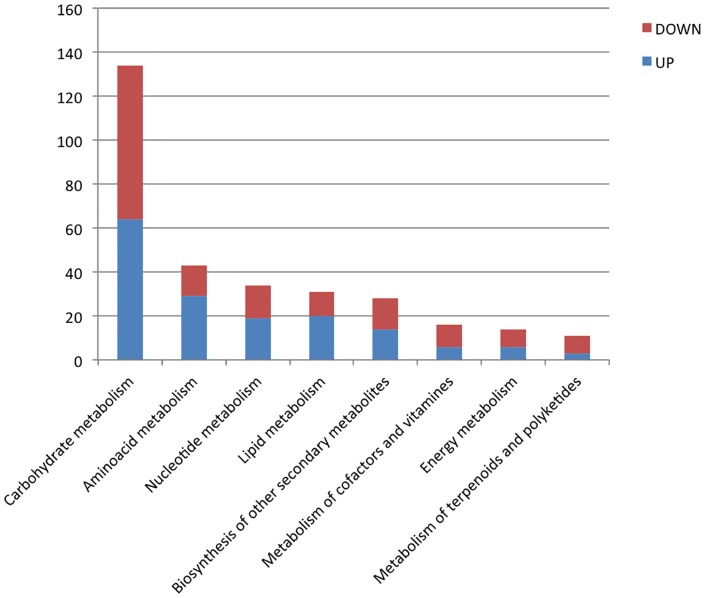
KEGG pathway assignment to differentially expressed genes in ECM roots versus non-symbiotic roots. The number of up and down transcripts predicted to belong to each category is shown.

To identify metabolic pathways in which DEGs were involved, pathway-based analysis was performed using the KEGG pathway database and BLAST2Go. As shown in Figure 2, ECM root colonization resulted in major alterations in the transcription of genes related to carbohydrate metabolism, followed by amino acid metabolism, nucleotide metabolism, lipid metabolism and biosynthesis of secondary metabolites, among others. These annotations provide a valuable recourse for investigating specific pathways involved in ECM symbiosis.

Quantitative Real-time PCR analysis was used to determine if the number of 454 reads obtained per transcript accurately reflected transcript levels in the analysed tissues. Analysis of a set of 454 highly expressed genes using quantitative RT-PCR in ECM root versus non-symbiotic root tissue confirmed the differential expression for all the tested genes (Fig. S6). The results confirmed the accuracy of our 454 generated data and validated pyrosequencing as an effective technology to quantify gene expression.

In the next sections we will discuss the results considering the genes found to be differentially expressed in cork oak roots upon ECM development and their putative role in the symbiosis. In this study, all differentially expressed genes (up- and down-regulated) were considered as putatively involved in the response of *Q*. *suber* roots to ECM symbiosis.

### Transcription factors responding to ECM colonization

Transcription factors (TFs) represent key proteins that regulate gene expression and as so, their regulation is indicative of the molecular mechanisms operating in a specific condition. TFs are highly conserved in eukaryotic organisms and are represented by various multigene families. In our study we identified many transcripts putatively encoding TFs some of them belonging to the same family (Table S3). The most abundant was the MYB TF family that has been reported to function in a variety of plant-specific processes, controlling development, metabolism and responses to abiotic and biotic stress [15]. Most of the transcripts representing this family were up-regulated in ECM cork oak roots, and included sequences related to the *tannin-related R2R3 MYB transcription factor* or the *MYB family transcription factor APL-like*. The first is involved in the regulation of proanthocyanidins (a flavonoid compound) biosynthesis in legumes [16]. The second is a TF required for phloem identity, having a dual role both in promoting phloem differentiation and in repressing xylem differentiation during vascular development [17]. Other families of TFs identified as differentially expressed in our study include members of the AP2/ERF superfamily, mostly down-regulated by the interaction with *P. tinctorius*, such as the *AP2-like ethylene-responsive transcription factor ail5-like*. This TF may be involved in the specification of meristematic or division-competent states in *Arabidopsis* young tissues [18]. The WRKY TFs family was also well represented. WRKY TFs are involved in regulating plants response to pathogens namely the plant innate immunity [19]. Altered transcription of WRKY TFs in our analysis is another indication of the existence of a common signaling pathway used by plants to respond to both pathogens and symbionts [20]. We also detected several members of the heat shock TFs that regulate the expression of heat shock genes that are activated by several abiotic stresses such as low temperature, osmotic stress, salt, oxidative stress, intense light and wounding [21]. Most of the heat shock TFs identified were up-regulated, a result that is in accordance with other studies reporting that ECM fungi induce transcription of abiotic stress related genes [5]. The transcriptional alteration of the above mentioned TFs in ECM roots is consistent with the notion that ECM fungi alter plant-specific cellular processes such as development, metabolism or responses to abiotic and biotic stresses.

### Candidate genes involved on ECM root architecture

Morphological observations of ECMs have shown that root architecture of the host plant is profoundly modified, with intense short root formation and cytodiferentiation of the colonized cells (radial elongation and root hair decay) [6], [22]. In our transcriptional analysis several genes involved in cellular processes occurring at root epidermal cells were regulated by the interaction and may constitute potential targets implicated in root hair inhibition in ECM roots. A sequence related to the *Arabidopsis GLABROUS1* (*GL1*) and *WERWOLF* (*WER*), which encode MYB-transcription factors that define cell fate in shoot and root epidermis [23] was found to be up-regulated in ECM cork oak roots. In *Arabidopsis,* epidermal cell fate specification (hair cell/non-hair cell) is determined by the competition between *WER* and *CPC* transcription factors required for promoting the non-hair cell fate and the hair cell fate, respectively [24]. It was found that over-expression of *WER* is sufficient to cause epidermal cells to adopt the non-hair cell phenotype [24]. Transcriptional induction of a transcript similar to *WER* in ECM cork oak roots strongly suggests that the host plant adopts a transcriptional mechanism for inhibiting root hair development involving a *WER*-signaling mediated process. Also, several transcripts reported to be involved in root hair growth via vesicle trafficking [25], such as *phosphatidylinositol kinase* sequences were differentially expressed. These proteins are involved in the production of phosphatidylinositol 4,5-bisphosphate, a lipid molecule that functions as a site specific signal on membranes to promote cytoskeletal reorganization and membrane trafficking in order to initiate and promote hair root growth. Down-regulation of these sequences in our study is consistent with root hair growth inhibition reported to occur upon ECM root colonization.

Hyphae of ECM fungi growing in the rizosphere induce an intense short-root formation, providing a means of increasing contact sites and niches for hosting the colonizing hyphae [26]. ECM fungi are able to produce ethylene and auxin that regulate plant root morphogenesis inducing primary root shortening and branching [27]. Felten et al. [4] showed that the ECM fungus *L. bicolor* stimulates lateral root formation in *P. trichocarpa* and *Arabidopsis* thought auxin transport and signaling. Our transcriptional analysis identified several DEGs involved in auxin-related processes (Table S4) many of them in common with the ones reported by Felten et al. [4]. These are genes known to play a role in auxin-induced lateral root formation [28] and include several *Aux/IAA* transcription factors and *auxin response factors* involved in auxin signaling, genes involved in auxin transport such as sequences related to the *auxin-induced protein 5NG4* and the *nodulin 21-like transporter*, and genes encoding enzymes involved in both auxin conjugation (*probable indole-3-acetic acid-amido synthetase -like*) and oxidation (*IAA-amino acid hydrolase IRL1-like 5-like*). The auxin signaling-related DEGs showed a heterogeneous expression profile. However, the ones related to auxin transport and auxin conjugation/oxidation were up-regulated, an indication of the negative feedback mechanism that regulates auxin homeostasis in root pericycle and the process of lateral root initiation [28]. Our results are in accordance with previous studies [29] and suggest that ECM fungal colonization induces an auxin transcriptional response that could be involved in the stimulation of lateral root initiation in ECM plants. Recently, it was reported that volatiles released by *L. bicolor* were sufficient to stimulate lateral root formation in *Arabidopsis* [30].

### ECM colonization affects flavonoid biosynthesis

**Figure 3:**
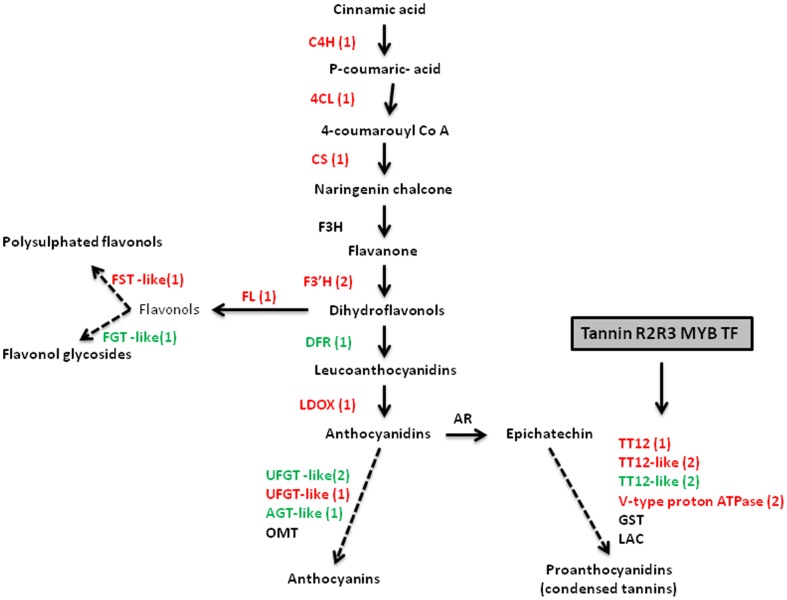
Predicted flavonoid pathway in cork oak ECM roots. Enzymes are indicated by capital letters; red color indicates transcripts that were up-regulated in ECM roots compared with non-symbiotic roots. Green indicates down-regulated transcripts. Numbers in brackets indicate the number of differentially expressed genes found in the present study. Dashed arrows indicate steps which are not yet fully understood. Grey box indicates transcriptional regulators. Abbreviations are as follows: *AGT* anthocyanidin -o-glucosyltransferase, *ANR* anthocyanidin reductase, *CHS* chalcone synthase, *C4H* cinnamate 4- hydroxylase, *4CL* 4-coumarate-CoA ligase, *DFR* dihydroflavonol 4-reductase, *F3 H* flavanone 3b-hydroxylase, *F3′H* flavonoid 3′-hydroxylase, *FST* flavonol sulphotransferase, *FGT* flavonol glucosyltransferase, *GST* glutathione S transferase, *LAC* laccase-like, *LDOX* leucoanthocyanidin dioxygenase, *OMT* O-methyltransferase, *tannin R2R3 MYB TF* tannin-related R2R3 MYB transcription factor, *V-type H^+^ ATPase* encoding a vacuolar proton pump that supports MATE antiporter, *TT12* transparent testa 12 enconding a multidrug and toxic compound extrusion-type (MATE) transporter, *UFGT* UDP-glucose:flavonoid-3-O-glycosyltransferase.

Flavonoids [flavonols, anthocyanins and proanthocyanidins (condensed tannins)] are aromatic amino acid-derived secondary metabolites with multiple functions in plant ecology and development, serving as pigments, protectants against biotic (pathogens) and abiotic stresses (UV light damage) and play an important role as signaling molecules in legumes where they serve as a chemoattractants for symbiotic nitrogen fixing bacteria [31]. Many of the enzymes involved in the flavonoid backbone biosynthesis were found to be differentially expressed in cork oak roots upon ECM inoculation (Figure 3; Table S5). Up-regulated sequences included chalcone synthase, the entry point enzyme of the flavonoid biosynthesis, flavonoid 3′-hydroxilase, flavonol synthase, the enzyme that leads to the production of flavonols and leucoanthocyanidin dioxigenase, the enzyme that leads to anthocyanins and proanthocyanidins. We also detected alterations in transcription of genes related to *PROTEIN TRANSPARENT TESTA 12*, a MATE transporter required for the vacuolar transport of proanthocyanidin precursors [32]. Additionally, sequences encoding regulators of flavonoid biosynthesis, such as tannin-related R2R3 MYB transcription factors were activated and repressed in our experiment. These transcription factors were shown to induce the biosynthesis and accumulation of proanthocyanidins through the up-regulation of late flavonoid biosynthetic genes and proanthocyanidin-specific MATE transporter [16]. Two transcripts encoding V*-type H^+^ ATPase* subunits were up-regulated and could be involved in providing a proton gradient across the vacuolar membrane to support the MATE transporter activity [32]. Overall the results seem to suggest an increased expression of the pathways that lead to flavonols and proanthocyanidins biosynthesis. In arbuscular mycorrhizas (AM), flavonoids have been shown to accumulate to significant levels in roots of a hypermycorrhizal *Medicago truncatula* mutant [33] and to stimulate growth of AM fungal hyphae *in vitro* [34]. In poplar ECM roots, Larsen et al. [35] detected a high enrichment for enzymes of the flavonoid metabolism and in an *in vitro* study Kiuchi et al. [36] showed that flavonoids induce the germination of ECM fungal spores suggesting that these compounds play a role as signaling molecules in ECM symbiosis. Our results are in good agreement with these reports and constitute an important resource to help defining the role of flavonoids in ECM symbiosis.

### ECM colonization induces cell wall remodeling

Our results point out to an extensive remodeling of the root cells walls in response to the contact with the ECM fungus, since many genes known to be involved in cell wall biosynthesis and remodeling were found to be differentially expressed (Table S6). The carbohydrate-active-enzyme (CAZymes) category was highly represented. Sequences putatively involved on glucan-chain elongation, like cellulose synthase-related genes and sucrose synthase, proteins involved in the cellulose synthase complex [37], were mainly up-regulated in ECM roots, suggesting an activation of cellulose synthesis. In contrast, several sequences related to the *callose synthase* gene were down-regulated. This result suggests a cell wall relaxation in ECM colonized roots since callose accumulation has been reported to be involved in reinforcement of the cell wall following pathogen attack [38]. Cell wall-related glycosyltransferases were mostly down-regulated which suggests an inhibition of the synthesis of non cellulosic polysaccharides in colonized root cells, since these enzymes are part of the biosynthetic machinery to synthesize the complex polysaccharides that are present in the plant cell wall, like pectin, hemicelluloses or xyloglucan [39]. On the contrary, cell wall-related glycosylhydrolases (e.g. polygalacturonase, endoglucanase, glucan endo-beta-glucosidase, xyloglucan endotransglycosylase-hydrolase, polygalacturonase) were mostly up-regulated. These enzymes are required for the modification of cell wall polysaccharides and are involved in wall loosening and elongation, formation of secondary cell walls of vascular tissues [40], as well as in the cleavage and/or rearrangement of the xyloglucan and pectin cell wall backbones [41], [42]. In addition, members of the carbohydrate esterase family were also identified as differentially regulated, such as pectinesterases which are described as being involved in modulation of cell wall stability [43]. Taken together, we hypothesize that these CAZymes are mediating the remodeling of root cell walls necessary to facilitate the progression of the symbiotic fungal hyphae into the apoplastic space of root cells during Hartig net development. In agreement, several class III peroxidases were mainly down-regulated. These extracellular oxido-reductases are secreted into the cell wall and have been described to reduce cell wall extensibility by forming covalent links between pectin residues [44]. Additionally, transcripts encoding expansins, proteins known to induce cell wall extension [45], were up-regulated. Supporting the activation of cell wall remodeling processes, genes encoding for wall-associated receptor kinases were up-regulated. These proteins are assumed to monitor changes in wall integrity and signal back to regulate the machinery involved in the synthesis and modification of the cell wall components, coordinating wall loosening and strengthening during cell expansion [46]. Concerning transport activity in the cell wall, we identified several up-regulated genes encoding cell wall-related transporters, namely *ADP-ribosylation factors* that are thought to be involved in vesicle trafficking to deliver new wall material, secretion of wall structural proteins, and delivery of new plasma membrane proteins for cellulose synthesis [47]. Altogether our results are indicative of an extensive alteration in the transcription of genes related to plant cell wall structure upon ECM development and are consistent with other studies reporting differential expression of plant cell wall genes upon ECM symbiosis [5], [10], [48]. The formation of the novel symbiotic interface must involve drastic cell wall remodeling to facilitate hyphal penetration between cells and formation of the Hartig net. Besides having to accommodate the symbiotic fungal hyphae, ECM colonized root cells also alter dramatically their architecture showing increased volume and radial elongation probably to increase the area for mutual nutrient exchange. During this process the cell wall must maintain its thickness probably through the addition of newly synthesized polysaccharides and proteins. Besides, ECM roots are highly ramified and the formation of new lateral roots implies the synthesis of new wall material and is accompanied by alterations in expression of cell wall-related genes [49].

### ECM colonization induces alterations in plant secretion

The secretory pathway comprises the endoplasmic reticulum (ER), Golgi apparatus, trans-Golgi network, pre-vacuolar compartment, vacuole and endosomes and is involved in the transport of vesicles that deliver proteins, polysaccharides and lipids to either the lysosomes/vacuoles or the plasma membrane [50]. Several genes putatively encoding proteins involved in the secretory pathway functioning altered their transcription in our transcriptomic analysis (Table S7). The proteins identified are involved in processes like formation of coated vesicles [51], budding of transport vesicles from the ER [52], protein translocation in the ER [53], [54], vesicular transport between the endoplasmic reticulum and the Golgi apparatus [55] or docking of exocytic vesicles with fusion sites on the plasma membrane [56], [57]. We also identified components of the ER-localized protein import and processing machinery, such as sequences related to components of the signal recognition particle (*signal recognition particle 54 kda protein 2-like*) and the signal peptidase complex (*signal peptidase complex catalytic subunit sec11c-like*, *signal peptide peptidase-like 1-like*, *probable signal peptidase complex subunit 1-like*) [50]. The majority of these sequences were up-regulated suggesting an activation of the secretory pathway in response to ECM symbiosis. In plant-microbe associations the secretory machinery seems to play a significant role in communication between plants and pathogens/symbionts required not only for the delivery of antimicrobial molecules, but also of cell surface sensors to detect microbes [50]. Recent studies highlighted the importance of the secretory pathway in plant root symbiosis by showing that in N_2_ fixing nodules, symbiosome (the functional homolog of ECMs hartig net) development is dependent on a gene encoding a subunit of the signal peptidase complex, and that upon rhizobial infection the plant machinery for processing secretory proteins is mobilized (up-regulated) coordinately [58]. An activation of genes involved in vesicle trafficking was also reported for ECM roots of *P. trichocarpa* [5]. In the context of ECM development an activation of genes involved in the secretory pathway is consistent with the alterations in expression of transcripts encoding proteins involved in cell wall remodeling, defense, and signaling events by secreted plant receptors (see below), observed in this and others studies [5], [10], [35], [48]. Interestingly, we also observed induction of a transcript encoding glutamate decarboxylase, the enzyme that catalyses decarboxylation of glutamate to γ -aminobutyric acid (GABA). In the mammalian nervous system GABA is the major inhibitory neurotransmissor and is released into the synapses via vesicular trafficking [59]. GABA is known to accumulate in leguminous plants being transferred from the host roots to the symbiotic bacteria possibly increasing N2 fixation by a presently unknown mechanism [60].

### Genes related to nutrient transfer

The essence of ECM symbiosis consists in the exchange of plant-derived carbohydrates for nutrients (e.g. nitrogen, phosphate) and water which occurs bi-directionally across the plant-fungus interface in the Hartig net [1]. Among the identified DEGs we found several sequences that can have a putative role in nutrient transfer between ECM symbiotic partners (Table S8). In ECM symbiosis up to 1/3 of plant photoassimilates can be transferred towards the fungal partner, host trees increasing their photosynthetic activity as a strategy to compensate the increased carbon sink promoted by the ECM fungus [3]. It is commonly accepted that plant sucrose, which enters via diffusion into the common apoplast of the plant/fungus interface, is hydrolyzed to sucrose and glucose by wall-bound plant invertases; glucose and fructose being substrates for specific fungal hexose transporters [3]. In our transcript profiling analysis we identified one *cell wall invertase* gene, up-regulated upon ECM root development that may be involved in directly delivering hexoses to the apoplast during symbiosis. We also identified one up-regulated transcript homologous of the *Arabidopsis cytosolic invertase 2* reported to be involved in the regulation of primary root elongation, root hair growth, and osmotic stress-induced inhibition of lateral root growth by controlling the concentration of hexoses in cells [61]. Induction of this transcript in ECM root symbiotic tissue could favor the supply of the increased energy demand of (colonized) root cells to fulfill their higher metabolite activity and structural changes. Also regulated by the symbiosis were genes involved in starch biosynthesis and breakdown, including *starch synthase* and *UDP-glucose pyrophosphorylase* (down-regulated), and *alpha-amylase* and *amidohydrolase* (up-regulated) related sequences. The transcript profile observed for these transcripts is consistent with an activation of sugars from cellular pools to promote and sustain symbiosis and has been also identified in other root symbiotic interactions [62]. ECM formation in cork oak roots altered the transcription of genes involved in plant glycolysis and TCA cycle. Six sequences related to the *6-phosphofructokinase* gene, one sequence encoding phosphoenolpyruvate carboxylase (PEPC) and another encoding a cytosolic malate dehydrogenase were all up-regulated, suggesting an activation of the glycolic pathway in ECM roots. In plants PEPC has a wide range of non-photosynthetic roles including supporting carbon-nitrogen interactions [63]. On the contrary, pyruvate kinase, another enzyme from the glycolytic pathway was down-regulated. PEPC and pyruvate kinase play a central role in the control of plant glycolysis and respiration since import of PEPC-derived malate into the mitochondria generally serves an anapleurotic role to support biosynthesis and nitrogen assimilation, whereas pyruvate derived from pyruvate kinase is the most significant substrate for respiration [63]. In symbiotic nodules, an interaction also characterized by an increased supply of N to the host plant, a legume specific PEPC isoenzyme is induced during active N_2_ assimilation, its suppression resulting in greatly diminished rates of nitrogen assimilation [64]. We observed a high level of expression for a transcript encoding carbonic anhydrase (CA), the enzyme responsible for the reversible hydration of CO_2_ to bicarbonate (HCO^−^_3_), one of the substrates of the PEPC-catalysed reaction. In symbiotic nodules, up-regulation of PEPC and CA were found to be involved on increased dark CO2 fixation for reincorporation of carbon lost during increased respiration for N fixation [65]. Reincorporation of CO_2_ can provide intermediates for the TCA cycle necessary for N assimilation. It is possible that a similar mechanism could be also operating in ECM roots in order to recycle the CO_2_ lost during respiration, since ECM plants should invest considerable amounts of carbon skeletons for assimilating N which is transferred by the fungus from the N poor forest soil. In accordance with the known role of ECM fungi in supplying N to the host plant [66], in our experiment genes associated to nitrogen assimilation, such as *nitrite reductase*, *glutamine synthetase*, a sequence related to the *nitrite transporter* that transfers N into the chloroplast for assimilation, and a chloroplastic-like ferredoxin-NADP reductase that provides reducing power for N assimilative enzymes, were all transcriptionally activated upon symbiosis.

Exchange of nutrients between plant and fungus relies on membrane transporters that are responsible for import and export of nutrients into the apoplastic space. Several sugar transporters responsible for the active uptake of sucrose were induced, which is in accordance with ECM plants higher photosynthetic activity that results from the increased sink activity of colonized roots. Concerning nitrogen transport, we detect differentially expressed ammonium and amino acid transporters, the two alternative N forms presumed to be translocated from the fungus to the plant during ECM interaction [66]. The only ammonium transporter detected was down-regulated upon root colonization and most of the amino acid transporters were also down-regulated. However, a sequence similar to a probable polyamine transporter, an amino acid transporter family protein, was strongly induced suggesting that amino acids could be indeed the preferentially N form to be translocated into the plant partner. In accordance with previous reports [35] inorganic phosphate transporters were repressed.

### Symbiont-recognition related genes

In the list of DEGs we detected many sequences related to proteins known to be involved in plant-microbe recognition and in plant's defense against pathogens (Table S9). The most abundant class was the disease resistance proteins of the NBS-LRR family (e.g. CC-NBS-LRR resistance proteins, TMV resistance proteins, NBS-LRR resistance proteins, NBS-containing resistance-like protein, disease resistance proteins RPM1-like proteins, disease resistance RPP8-like proteins). These proteins are involved in innate immunity by detecting a diverse array of pathogens including bacteria, viruses, fungi, nematodes, insects and oomycetes [67]. They act by monitoring the status of plant proteins targeted by pathogen effectors (membrane receptors) triggering the plant defense system including the hypersensitive response, which restricts pathogen growth. The great variety of NBS-LRR proteins identified in our study suggests a complex signaling mechanism where plant cells activate and repress many types of “sensor” proteins in response to ECM colonization. Up-regulated sequences in our study are probably implicated in recognition events during the initial phases of ECM interaction, whereas down-regulated sequences could be related to mature stages, since a repression of defense-related genes has been detected in mature stages of ECM symbiosis in several plants [10], [48]. How ECM fungi escape/repress the host innate immunity system is still unknown but it could involve suppression of salicylic acid (SA) response the major signaling pathway induced during pathogenic interactions [68]. Accordingly, we detected a down-regulated NPR1-1 homolog, a protein that senses and transduces salicylic acid (SA) and is involved in the signaling pathway that leads to local and systemic defense responses in pathogenic interactions [69]. This result suggests an inhibition of SA-mediated defense response in ECM symbiotic roots, probably for allowing the penetration of fungal hyphae in the apoplast and formation of the Hartig net. In *Medicago truncatula* transient over-expression of *NPR1* was shown to have a negative effect on symbiotic nodule establishment by interfering with root hair deformation, essential to sequester the symbiotic bacteria, while inhibition of *NPR1* had a positive effect and accelerated root hair curling [70]. Also, quitinase related sequences, known to possess anti-fungal activity by degrading fungal cell walls, were repressed in our experiment. Other interesting genes identified, to which an active role in pathogen defense has been ascribed include the *brassinosteroid insensitive 1-associated receptor kinase 1* (*BAK1*), and the *LRR receptor-like serine threonine-protein kinase FLS2-like* (*LRR-RLK FLS2-like; flagellin sensing 2-like*). In our study, *BAK 1*was one of the most highly expressed genes and a sequence related to the *LRR-RLK FLS2* gene was also highly expressed. These are plasma membrane receptor kinases containing an extracellular domain that recognizes and binds fungal and bacterial β-1,4-linked amino sugars, such as chitin and peptidoglycans [71], [72]. Binding to the receptor is the first step to initiate an intracellular MAP kinase signaling cascade that results in a coordinated defense response, although the details of such signal transduction pathway are not yet fully understood. The role of these two membrane receptor proteins in defense is thought to be associated with their mutual interaction in the plasma membrane during the early signal transduction pathway that activates the defense program upon pathogen infection, resulting in enhanced resistance [71]. It was found that specific effector proteins from pathogenic bacteria directly target *BAK1*, and interfere with the formation of the *FLS2*/*BAK1* complex blocking the downstream plant immune response [72]. Recent studies show that pathogenesis related proteins are also involved in the signaling pathway that mediates plant interactions with fungi in AM symbiosis [20]. Also, in ECM symbiosis, a secreted effector protein (MiSSP7) from *L. bicolor*, resembling the effectors from pathogenic fungi and bacteria was found to be required for symbiosis development [73]. Other identified transcripts encoding signal transduction components found, include the *nodulation receptor kinase* (*NORK*) and the *ion channel DMI1*. The *NORK* gene encodes an LRR receptor-like kinase that is predicted to function in the Nod factor perception/transduction that initiates a signal cascade leading to nodulation [74]. The family of ‘NORK extracellular-sequence-like’ (NLS) genes is broadly distributed in the plant kingdom and is presumed to function in the perception and transduction systems for extracellular ligands, the rhizobial Nod factor, leading to development of the symbiotic root nodule [74]. The altered expression of membrane receptor proteins upon ECM development is in accordance with the observed increased transcription of genes related to the secretory pathway activity detected in our study. Interestingly, one of the most up-regulated genes in our experiment was a homolog of the ion channel *DMI1* which is required for the early signal transduction events leading to endosymbiosis (called the SYM pathway). It encodes a nuclear cation channel that is necessary for the initiation of Nod and Myc factor-induced calcium spiking during symbiotic signaling [75]. Induction of transcripts related to *NORK* and *DMI1* genes of legumes in our study suggests the existence of a common signal transduction pathway operating during the establishment of symbiosis with endosymbionts and ectosymbionts. There is a great lack of knowledge on the signal perception mechanisms that are responsible for ECM symbiont recognition in plant cells. The data presented here highlight the similarity between symbiotic and pathogenic plant signals. According to recent studies, symbiotic fungi evolved from saprophytic ancestors that through convergent evolution developed the ability to establish symbiosis with plants [76]. Our results in ECM symbiosis and reports on other root symbiosis suggest that plants have adopted the “same” proteins to respond to pathogens and symbionts, with limited alterations in specific domains of those proteins having played a crucial role in shifting the intracellular signaling response from defense to symbiosis [77]. Genes related to *BAK 1*, *LRR-RLK FLS2-like*, *NORK*, *DMI1*, among others, identified in our study constitute potential targets for the recognition events between the root cells and ECM fungi. It would be interesting to investigate if these and other receptor kinases detected in our experiment could interact with ECM fungal effectors like MiSSP7.

## Conclusion

In this study we used 454 sequencing to investigate the transcriptional response of a host root to the establishment of ECM symbiosis. Analysis of the DEGs suggest an extensive remodeling of the cell wall, activation of the plant secretory pathway, increased biosynthesis of flavonoid compounds and expression of a great variety of genes involved in fungal recognition. In addition, we identified genes with putative roles in ECM-associated alterations in root architecture, such as stimulation of lateral root formation and root hair decay, and genes involved in nutrient exchange between symbiotic partners. Many of the plant genes identified in our transcriptional analysis were found to be homologous to genes previously identified in symbiotic root nodules and arbuscular mycorrhizas, including homologous of the common SYM genes, suggesting that similar pathways are activated by endosymbionts and ectosymbionts. The resources developed in this study provide an important tool for researchers in plant root symbiosis and constitute a foundation for future analysis to further increase our understanding on the molecular mechanisms underlying ECM symbiosis.

## Materials and Methods

### Ethics statement

Cork oak seeds were collected from trees located in the north (Braga; N 41°48'50.78'', O 6°47'30.98'') and center (Lisboa; N 38° 44' 4.69'', W 9° 10' 44.41'') of Portugal. No specific permissions were required for collecting seeds in these locations; this activity did not involved endangered or protected species.

### Production of cork oak ECM plants

*Pisolithus tinctorius* (strain Pt23 in the collection of the Center for Biodiversity, Functional & Integrative Genomics, Sciences Faculty of Lisbon University) was grown on BAF agar medium and subsequently in a peat-vermiculite mixture moistened with liquid BAF medium as described previously [14]. Seeds were germinated in a greenhouse, in plastic trays containing soil acquired from a gardening store (Siro Universal, Portugal; 80–150 mg/L N, 80–150 mg/L P_2_O_5_, 300–500 mg/L K_2_O, pH (CaCl_2_) 5.5–6.5, organic matter >70%). After germination, three months old plantlets were transferred to 1,5 L pots containing soil and simultaneously inoculated with *P. tinctorius* peat-vermiculite inoculum (3 months old), according to Sebastiana et al. [14]. For the control treatment, cork oak plantlets were treated with a non-inoculated peat-vermiculite mixture. Plants were grown in pots in a greenhouse and watered once a week with 500 ml of tap water. No fertilization was applied. Mycorrhizal roots started to be visualized 3 weeks after inoculation. Because we intended to cover as much as possible the entire ECM developmental process, plants were harvested at one, three, eight and sixteen weeks after *P. tinctorius* inoculation, in order to include early and mature stages of interaction. Non-inoculated control plants were harvested at the same time-points. The roots of 10 plants were collected for each treatment at each time-point after inoculation. Roots were rinsed with tap water to remove soil particles, and secondary roots were immediately frozen in liquid nitrogen and subsequently stored at −80°C.

### cDNA library construction and pyrosequencing

In order to compare gene expression between mycorrhizal and non-symbiotic conditions two separate cDNA libraries were prepared for pyrosequencing: one from mycorrhizal roots and another from non-symbiotic roots. Each library was composed of a pool of root tissue from 40 plants (10 plants per post-inoculation time-point). Firstly, total RNA was extracted from each time-point sample using the hot borate method procedure [78]. RNA quality was checked on 1% agarose gels, and quantity was measured on a NanoDrop ND 1000 spectrophotometer (Thermo Scientific, FL). The RNA obtained from each time-point sample was subsequently pooled and used in cDNA synthesis and pyrosequencing. Briefly, RNA preparations were assessed for quality with the RNA pico 6000 kit (Agilent Technologies, Waldbronn, Germany) and 2100 Bioanalyser (Agilent Technologies) and for quantity by fluorescence with the Quant-iT Ribogreen RNA Assay kit (Invitrogen, CA, USA). Poly(A) RNA was isolated from total RNA (50 µg) using the MicroPoly(A) Purist Kit (Ambion). Poly(A) RNA (200 ng) was fragmented and used as template for double stranded cDNA production using random hexamer primers according to the procedure of the cDNA System Synthesis Kit (Roche). ECM and control cDNA samples were submitted to the Sequencing Advanced Services Unit at Biocant (Portugal) for pyrosequencing, each in a full plate of 454 GS FLX Titanium according to standard manufacturer's instructions (Roche-454 Life Sciences, Brandford, CT, USA). Sequence reads were deposited in the NCBI Sequence Read Archive (SRA) under the accession number SRA106173.

### Sequence processing, assembly and annotation

Following 454 sequencing, reads were processed using SeqClean (DFCI Gene Indices Software Tools, http://compbio.dfci.harvard.edu/tgi/software/) to remove low quality reads and adaptor/primer sequences. Since we expected a lot of sequences from *P. tinctorius* (the ECM fungus used for ECM establishment) and from other soil organisms, since plants were grown in pots in a greenhouse, we applied several filters using SeqClean in order to eliminate reads from those organisms before the assembling process. First, reads were screened by searching against *P. tinctorius* transcriptome sequences retrieved from the JGI portal (http://genome.jgi.doe.gov/programs/fungi/ind) and against all bacteria, fungi and viral genome sequences downloaded from Gen-Bank (http://www.ncbi.nlm.nih.gov). Furthermore, reads that mapped to the *Arabidopsis thaliana* mitochondrion and chloroplast genome (http://www.Arabidopsis.org/) sequences were trimmed. The processed 454 reads were then assembled using MIRA assembler (version 3.0.0rc4) [79], run in its “accurate” mode with the assembly type set as “EST”. Due to the expected a high degree of “contaminating” organisms in our cDNA libraries, as explained above, sequences were taxonomy annotated in order to select only sequences belonging to the Streptophyta phylum, putatively originating from cork oak. To obtain taxonomic information we submitted the assembled sequences to MG-RAST [80], an automated analysis platform for metagenomes that provides taxonomical assignments based on sequence similarity to both protein and nucleotide databases. Since MG-RAST is optimized for the analysis of prokaryotic sequence data [80] we also searched our sequence dataset against NCBI. First, the contigs were subjected to BLASTX [81] (E value ≤e^−6^) against NCBI nr protein database. Second, files were filtered for redundancy in protein accessions. Unique contigs annotated as Streptophyta were selected for further analysis. Finally, contigs annotated as Streptophyta by the two procedures described above were considered to represent a set of unique cork oak root genes and were selected for further analysis. Functional annotation was performed using the BLAST2Go program [82] with BLASTX search against NCBI nr protein database (E value ≤e^−6^) and HSP length cutoff of 33. The unigene set was further classified into functional categories using the plant-specific GO slims in BLAST2Go. In order to evaluate the quality of our transcript set BLASTN searches (E value ≤e^−6^) were carried out against oak 454 EST sequences from the TIGR gene indices portal (release 2.0) (downloaded from http://compbio.dfci.harvard.edu/tgi/) and from the Fagaceae Genomics Web portal (downloaded from http://www.fagaceae.org).

### Identification of Differentially Expressed Transcripts

Differentially expressed genes were identified as genes showing significant higher/lower expression levels in symbiotic root tissue versus non-symbiotic root tissue. The number of reads mapping to each unigene in the two tissues analyzed (symbiotic and non-symbiotic) was counted and used as an approximate estimation of gene expression level in the corresponding tissues. For unigenes with multiple transcripts we summed the number of reads in each tissue and assumed that to be the corresponding final read count. Read counts were normalized using the EDASeq package [83]. EDAseq performs within-lane normalization, followed by between-lane normalization of the gene expression levels. With these procedures we reduced the gene length bias leading to more accurate estimates of expression fold-changes and tests of differential expression. Statistical significance of the differential gene expression between ECM and non-symbiotic roots was determined using the edgeR Bioconductor software package [84]. edgeR examines differential expression of replicated count data, by considering an overdispersed Poisson model in order to account either for biological and technical variability. In order to overcome the so-called overdispersion problem edgeR proposes to model count data with a negative binominal distribution. In accordance with the edgeR user's guide, since our data did not have biological replicates from which to estimate biological variability, we picked a reasonable dispersion value by considering the common square-root dispersion (biological coefficient variation) to be 0.1 (data on genetically identical model organisms arising from well-controlled experiments), a typical value reported in mRNA-seq studies with next-generation sequencing [85]. Once the negative binominal models were fitted to both conditions tested, edgeR used a generalized linear model (glm) likelihood ratio test for determining differential expression. The resulting row p-values were corrected for multiple tests according to Benjamini and Hochberg's [86] method. Genes were considered differentially expressed if exhibiting at least an adjusted p-value ≤0.001 and an absolute log_2_ (FC) ≥2.

### Quantitative real-time PCR confirmation

cDNA was synthesized from 2.5 µg of total RNA from ECM roots and non-symbiotic roots using RevertAidH Minus Reverse Transcriptase (Fermentas, Ontario, Canada) and Oligo(dT)_23_ primer (Sigma Aldrich), according to manufacturer's instructions. Gene specific primers were designed using Primer Express (version 3) software (Applied Biosystems) and the specificity of each primer pair was checked by BLASTN against all the 454 assembled sequences. Primer sequences for the genes used are listed in Table S10. Real-time PCRs were performed using diluted cDNA (1∶10), 0.2 µM of gene specific primers, 2.5 mM MgCl_2_ and Maxima SYBR Green qPCR Master Mix (2×) kit (Fermentas) using a StepOne Real-Time PCR system (Applied Biosystems). Thermal cycling for all genes started with a denaturation step at 95°C for 10 min followed by 45 cycles of denaturation at 95°C for 15 s and annealing temperatures for 30 s (Table S10). Each set of reactions included no template control and four technical replicates were done. The specificity of the PCR reactions was verified by melting curve analysis. The amplification efficiency (*E*) of each gene was determined using a pool of identical volumes of ECM root and non-symbiotic root cDNA samples. The pool was used to generate a five-point standard curve based on a ten-fold dilution series. Amplification efficiency was calculated from the slope of the standard curve (E = 10^(−1/a)^) where *a* is the slope of the linear regression model (y = a log(x)+b) fitted over log-transformed data of the input cDNA concentration (y) plotted against quantification cycle (Cq) values (x). A gene coding for a Tubulin γ-1 chain (qs_c4154), which revealed no alteration in expression between ECM and non-symbiotic roots in our 454 sequencing data, was used as reference to normalize qPCR expression. To calculate gene expression, the 2^−ΔΔCT^ method [87] was used.

## Supporting Information

Figure S1 **The ectomycorrhizal symbiosis between** ***Quercus suber*** **roots and** ***P*** **.** ***tinctorius*** **.** (a) Non-inoculated roots. (b) Colonized lateral root, 3 weeks after inoculation; fungal hyphae are starting to unsheathe the root tips. (c) Colonized lateral root, 8 weeks after inoculation; the fungal mantle is completely developed. (d) Microscopy image of a transverse section of a colonized root tip, 8 weeks after inoculation, showing the mantle (m), and the hartig net (hn) around epidermal root cells; scale, 50 µm.
(TIF)

Figure S2 **Length distribution of total number of high-quality reads generated.**
(PNG)

Figure S3 **Length distribution of total number of contigs generated.**
(PNG)

Figure S4 **Ranking of phylum abundances in the assembled 454 data annotated by MGRAST.** The y-axis plots the abundances of annotations in each phylum on a log scale.
(TIF)

Figure S5 **Top hit species distribution among the BLAST results of cork oak root unigenes against NCBI non redundant database.**
(PNG)

Figure S6 **Relative expression levels of selected cork oak transcripts validated by real-time PCR.**
(DOCX)

Table S1 **Annotated unigenes expressed in ECM and non-symbiotic cork oak roots.**
(XLSX)

Table S2 **Up- and down-regulated genes in ECM roots when compared to non-symbiotic roots.**
(XLSX)

Table S3 **Differentially expressed genes putatively encoding transcription factors.**
(XLSX)

Table S4 **Differentially expressed genes putatively encoding auxin-related genes.**
(XLSX)

Table S5 **Differentially expressed genes putatively encoding flavonoid-related genes.**
(XLSX)

Table S6 **Differentially expressed genes putatively encoding cell wall-related genes.**
(XLSX)

Table S7 **Differentially expressed genes putatively encoding secretory pathway-related genes.**
(XLSX)

Table S8 **Differentially expressed genes putatively encoding symbiotic nutrient transfer-related genes.**
(XLSX)

Table S9 **Differentially expressed genes putatively encoding symbiont recognition-related genes.**
(XLSX)

Table S10 **Target genes for qPCR analysis: primer sequences, amplicon length, PCR efficiency, annealing (Ta) and melting (Tm) temperatures.**
(DOCX)
