# Supplementary figures and images for: Oak Root Response to Ectomycorrhizal Symbiosis Establishment: RNA-Seq Derived Transcript Identification and Expression Profiling
Source: PLoS One. 2014 May 23;9(5):e98376. doi: 10.1371/journal.pone.0098376 (PMC4032270; doi:10.1371/journal.pone.0098376)

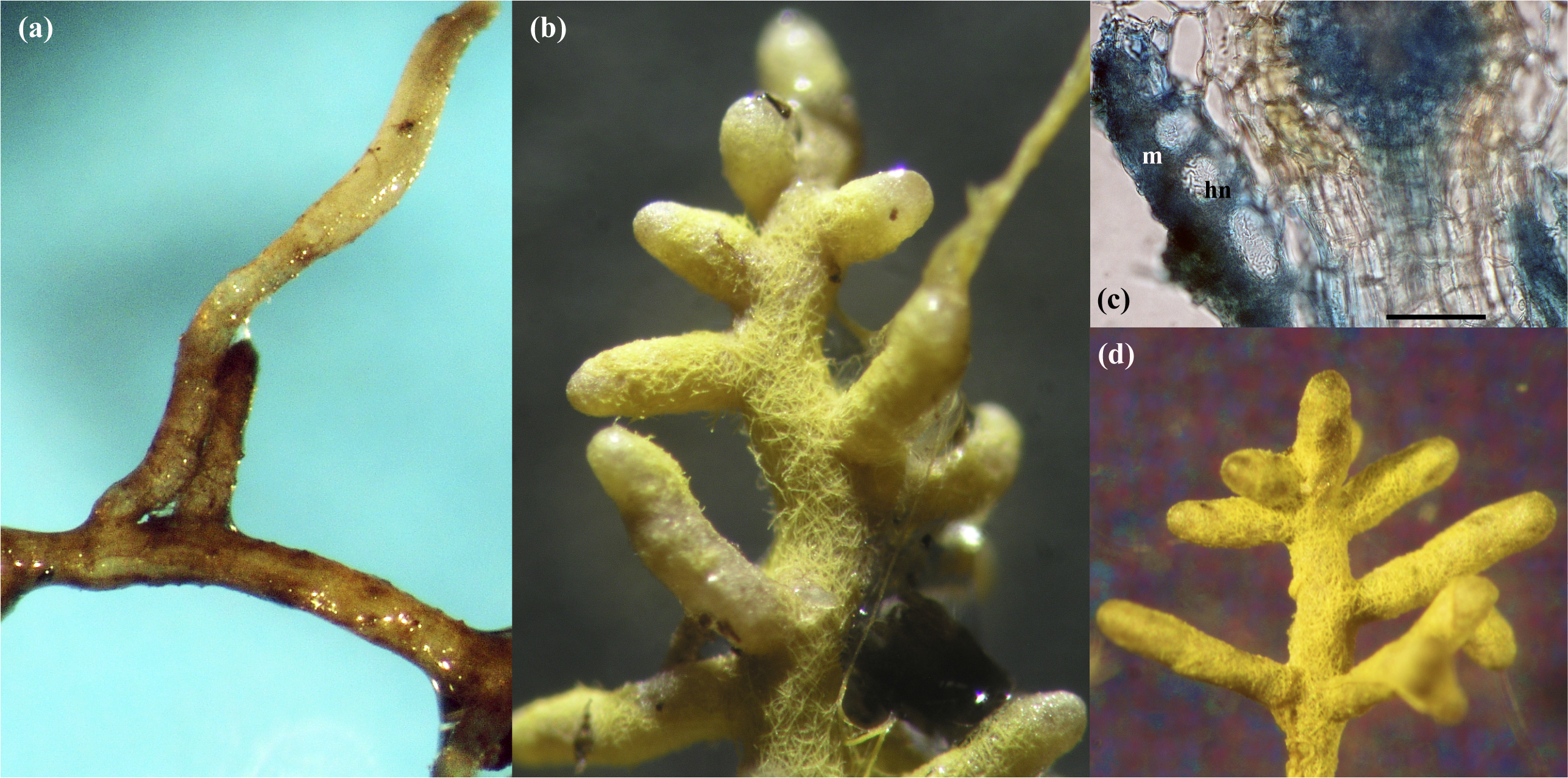

Supplement: Figure S1 — The ectomycorrhizal symbiosis between Quercus suber roots and P . tinctorius . (a) Non-inoculated roots. (b) Colonized lateral root, 3 weeks after inoculation; fungal hyphae are starting to unsheathe the root tips. (c) Colonized lateral root, 8 weeks after inoculation; the fungal mantle is completely developed. (d) Microscopy image of a transverse section of a colonized root tip, 8 weeks after inoculation, showing the mantle (m), and the hartig net (hn) around epidermal root cells; scale, 50 µm. (TIF) [file pone.0098376.s001.tif]

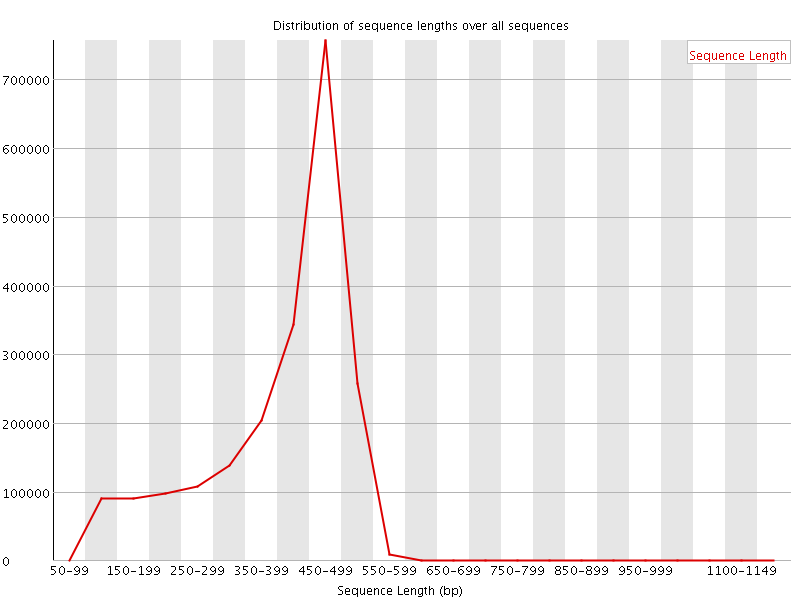

Supplement: Figure S2 — Length distribution of total number of high-quality reads generated. (PNG) [file pone.0098376.s002.png]

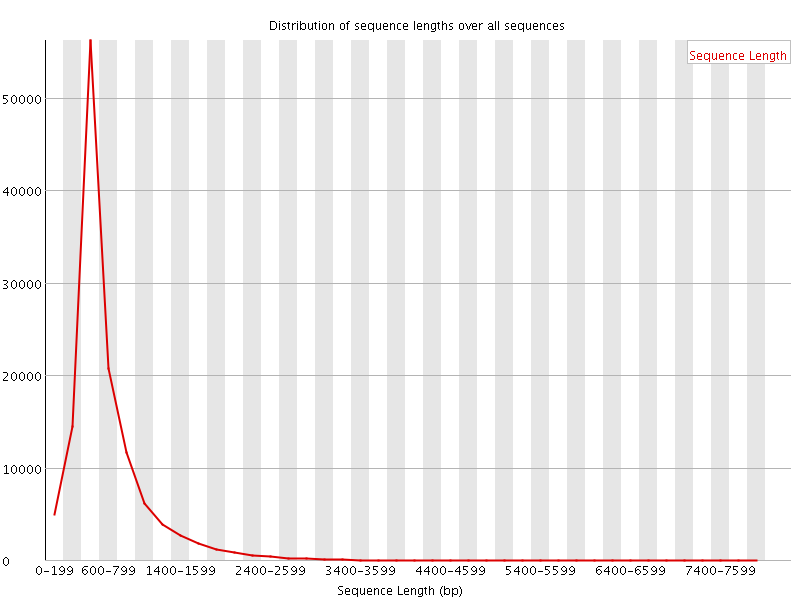

Supplement: Figure S3 — Length distribution of total number of contigs generated. (PNG) [file pone.0098376.s003.png]

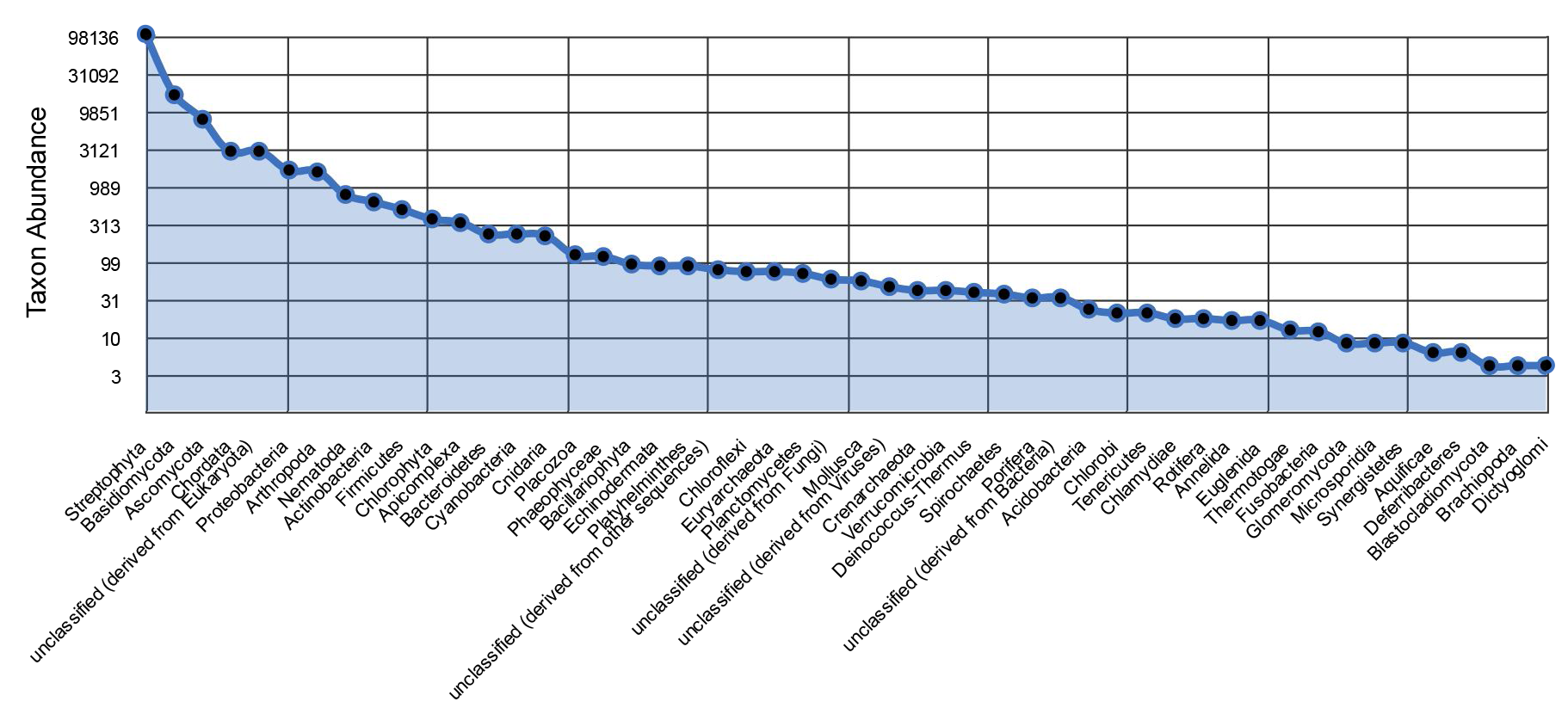

Supplement: Figure S4 — Ranking of phylum abundances in the assembled 454 data annotated by MGRAST. The y-axis plots the abundances of annotations in each phylum on a log scale. (TIF) [file pone.0098376.s004.tif]

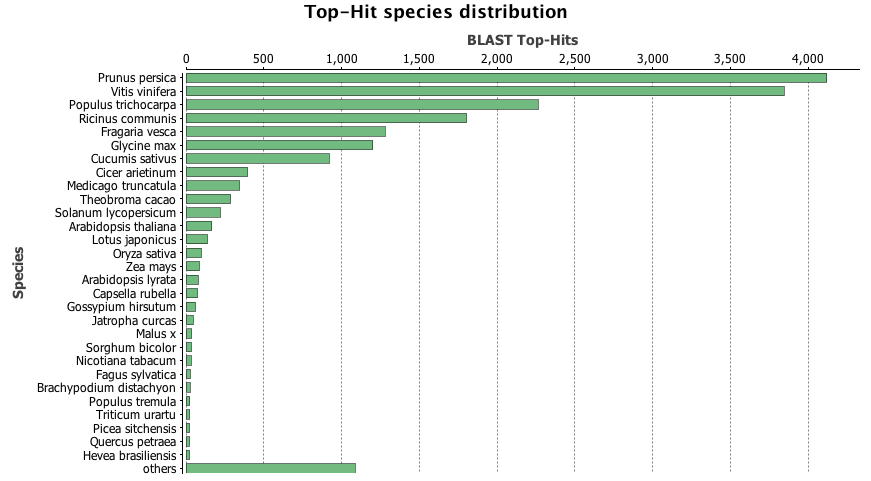

Supplement: Figure S5 — Top hit species distribution among the BLAST results of cork oak root unigenes against NCBI non redundant database. (PNG) [file pone.0098376.s005.png]
