# Supplementary material for: Oak Root Response to Ectomycorrhizal Symbiosis Establishment: RNA-Seq Derived Transcript Identification and Expression Profiling
Source: PLoS One. 2014 May 23;9(5):e98376. doi: 10.1371/journal.pone.0098376 (PMC4032270; doi:10.1371/journal.pone.0098376)
Supplement: Table S10 — Target genes for qPCR analysis: primer sequences, amplicon length, PCR efficiency, annealing (Ta) and melting (Tm) temperatures. (DOCX) [file pone.0098376.s016.docx]

| **ID** | **BLAST2Go annotation** | **Primer sequence (5’-3’)** | **Amplicon length (bp)** | **Ta (ºC)** | **Tm (ºC)** | **PCR efficiency (%)** |
| --- | --- | --- | --- | --- | --- | --- |
| qs_c4154 | Tubulin γ-1 chain | F: ATTGCTGATCGACCTTGAG | 142 | 55 | 76.43 | 93 |
|  |  | R: CCCCTGATGATAACCACT |  |  |  |  |
| qs_rep_c44924 | Early-responsive to dehydration stress-related protein | F: CTGAACTTGAAAGGCTATC | 121 | 55 | 73.77 | 93 |
|  |  | R: TGGCCACAATCACACTCT |  |  |  |  |
| qs_c10825 | BRASSINOSTEROID INSENSITIVE 1-associated receptor kinase 1 | F: AAAGTTCCGTGCCAAACTA | 94 | 52 | 77.60 | 103 |
|  |  | R: AGGGGTGCCAGCAACAA |  |  |  |  |
| qs_c30214 | 1-aminocyclopropane-1-carboxylate synthase | F: AAGCGGTGAGCGAGGAGAC | 139 | 60 | 76.67 | 91 |
|  |  | R: AAGGATCCGAGTATTTGTAGA |  |  |  |  |
| qs_c56518 | ARM repeat protein interacting with ABF2 | F: CGTCCATGAATCTTCTCCTC | 111 | 60 | 81.13 | 90 |
|  |  | R: TATCAAGGTTGGCGGTGTTC |  |  |  |  |
| qs_c2509 | Carbonic anhydrase 2 | F: GTTTGGGGCCATCGTCTG | 118 | 60 | 78.99 | 101 |
|  |  | R: TTCTGCTGTGGAGTATGCTGTTT |  |  |  |  |
| qs_c8287 | Clathrin light chain 2-like | F: GGCAATAGAGAAAAAGGGGA | 146 | 56 | 78.75 | 92 |
|  |  | R: TTCATATGGGGAGGAGGA |  |  |  |  |
| qs_c12983 | Cell wall invertase | F: AGTGGCCAATCAAGGAAATAG | 115 | 60 | 76.30 | 93 |
|  |  | R: TCGGCCTGTGAAGCTGTAAT |  |  |  |  |
| qs_c16126 | IAA-amino acid hydrolase ILR1-like 5 | F: CCACCTCTGACATAAGTA | 133 | 55 | 78.08 | 93 |
|  |  | R: GCTGCAGATCCCCATAAAA |  |  |  |  |

**Table S10 Target genes for qPCR analysis: primer sequences, amplicon length, PCR efficiency, annealing (Ta) and melting (Tm) temperatures**
