# Supplementary material for: Oak Root Response to Ectomycorrhizal Symbiosis Establishment: RNA-Seq Derived Transcript Identification and Expression Profiling
Source: PLoS One. 2014 May 23;9(5):e98376. doi: 10.1371/journal.pone.0098376 (PMC4032270; doi:10.1371/journal.pone.0098376)
Supplement: Supplementary file 12 [file pone.0098376.s006.docx]

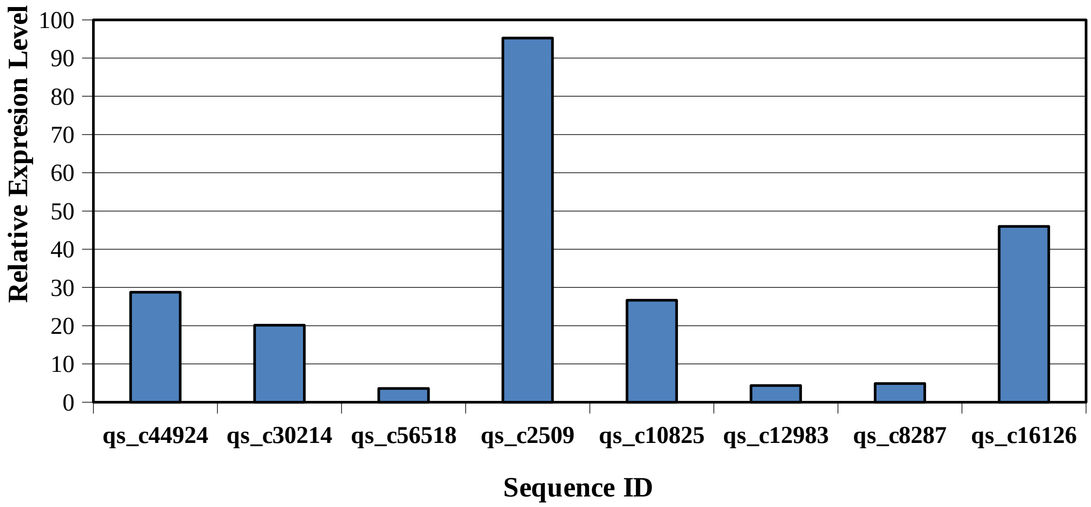
Relative Expresion Level
100
90
80
70
60
50
40
30
20
10
0
qs_c44924
qs_c30214
qs_c56518
qs_c2509
qs_c10825
qs_c12983
qs_c8287
qs_c16126
Sequence ID
